# An Unclassifiable Malignant Ovarian Germ Cell Tumor With Focal Choriocarcinoma-Like Features: A Case Report With Diagnostic Ambiguity and Adjuvant Treatment Considerations

**DOI:** 10.7759/cureus.109918

**Published:** 2026-05-30

**Authors:** Tadahisa Takeuchi, Hiroyasu Nakajima, Natsuko Hirose, Hachidai Hirakawa, Takahiro Kusaba

**Affiliations:** 1 Department of Gynecology, Oita Prefectural Hospital, Oita, JPN; 2 Department of Pathology, Oita Prefectural Hospital, Oita, JPN

**Keywords:** adjuvant chemotherapy, bleomycin etoposide cisplatin, choriocarcinoma-like features, ovarian germ cell tumor, unclassifiable tumor

## Abstract

Malignant ovarian germ cell tumors (MOGCTs) are uncommon neoplasms with substantial histologic diversity. Although most tumors can be assigned to established subtypes, some tumors show overlapping morphologic and immunophenotypic findings that complicate classification and management. We report a 37-year-old nulligravid woman with a right ovarian tumor, massive ascites, and elevated serum human chorionic gonadotropin (hCG). Histopathological examination revealed heterogeneous findings, including focal yolk sac tumor-like morphology and focal choriocarcinoma-like differentiation. Tumor cells were positive for AE1/AE3 and glypican-3, while AFP, SALL4, OCT4, and CD30 were negative; scattered cells were positive for hCG, GATA3, and p57. The tumor was diagnosed as an unclassifiable malignant ovarian germ cell tumor, International Federation of Gynecology and Obstetrics (FIGO) stage IC1. A key management issue was the postoperative intensity of bleomycin, etoposide, and cisplatin (BEP) chemotherapy. Because of incomplete formal staging and persistent histologic ambiguity, four cycles of BEP were selected rather than treatment de-escalation. The patient remains disease-free on follow-up. This case highlights the following two practical lessons: unusual ovarian germ cell tumors may remain unclassifiable despite integrated pathology, and adjuvant treatment decisions in stage I disease may depend not only on stage but also on staging adequacy and biologic uncertainty.

## Introduction

Malignant ovarian germ cell tumors (MOGCTs) are rare ovarian neoplasms that typically affect adolescents and young women and are usually highly sensitive to platinum-based chemotherapy [1-4]. Most tumors can be classified as dysgerminoma, yolk sac tumor, immature teratoma, embryonal carcinoma, choriocarcinoma, or mixed germ cell tumor [5]. In routine practice, however, some ovarian germ cell tumors show overlapping histologic and immunophenotypic findings that do not fulfill criteria for a single well-defined subtype.

When classification remains uncertain, management can become difficult. Postoperative treatment decisions in stage I disease are increasingly individualized, particularly as surveillance and treatment de-escalation have become accepted for selected low-risk ovarian germ cell tumors [6-10]. Yet these frameworks are harder to apply when staging is incomplete or the tumor has biologically ambiguous features.

We report a case of stage IC1 unclassifiable malignant ovarian germ cell tumor with focal choriocarcinoma-like features. This case is educational because it illustrates both a diagnostic pitfall and a practical question in adjuvant management - whether treatment de-escalation is appropriate when formal staging is incomplete and pathology remains indeterminate.

## Case presentation

**Figure 1:**
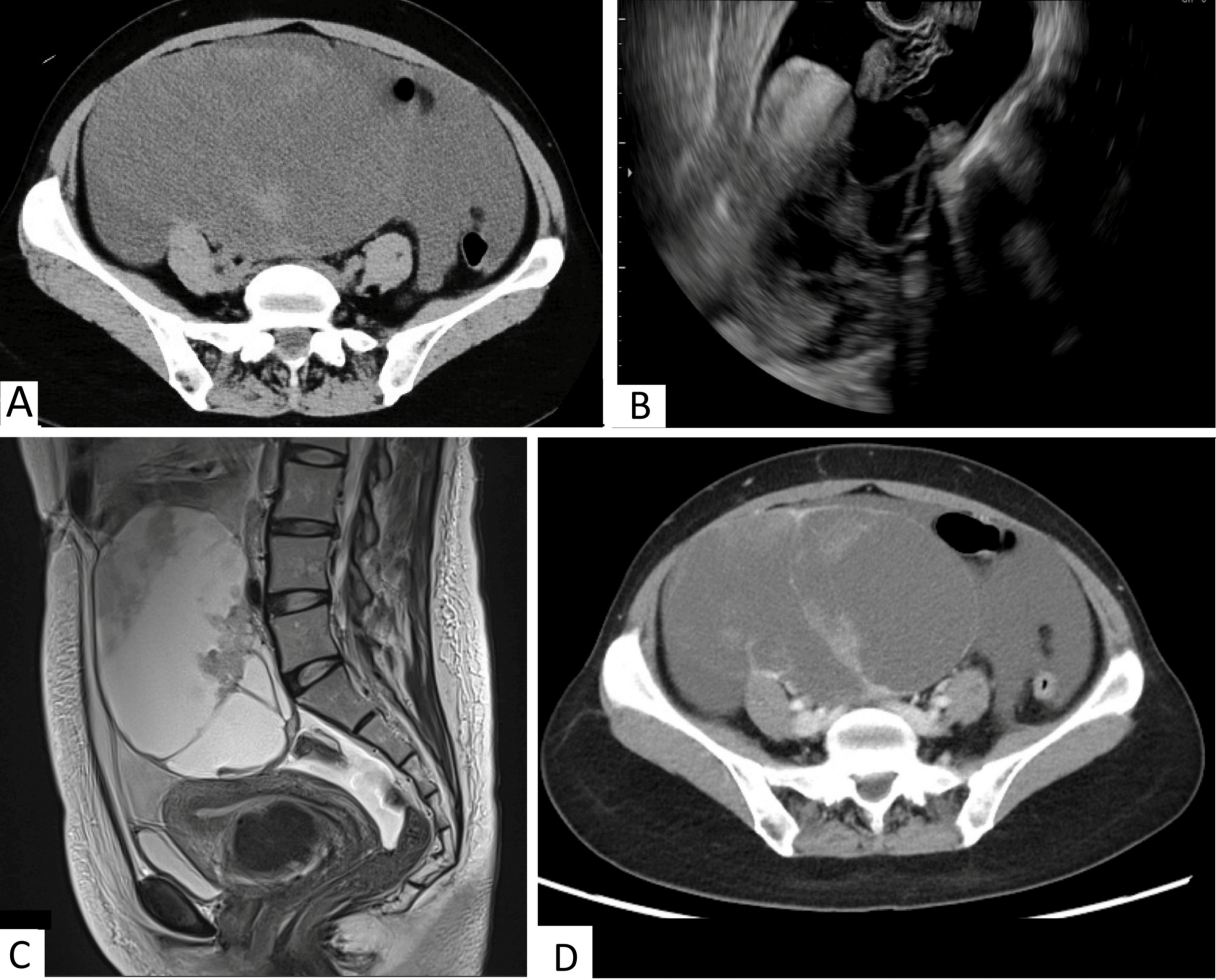
Preoperative imaging findings in a 37-year-old woman with elevated serum hCG showing a right ovarian mass and massive ascites. (A) Non-contrast computed tomography at the referring facility showed a large right adnexal mass and intraperitoneal fluid accumulation. (B) Transvaginal ultrasonography demonstrated a multilocular cystic mass arising from the right adnexa. (C) Pelvic magnetic resonance imaging demonstrated a 15-cm multilocular cystic mass arising from the right ovary with focal diffusion restriction. (D) Contrast-enhanced computed tomography confirmed the large right ovarian mass with massive ascites and showed no lymphadenopathy or distant metastases. hCG: human chorionic gonadotropin

A 37-year-old nulligravid woman with schizophrenia requiring long-term psychiatric hospitalization presented to a local emergency department with a two- to three-week history of abdominal pain. A qualitative urine pregnancy test was positive. Non-contrast computed tomography (CT) showed a right adnexal mass and intraperitoneal fluid accumulation, and serum human chorionic gonadotropin (hCG) was elevated at 900.8 mIU/mL (reference range: <5.0 mIU/mL in non-pregnant women) (Figure 1, panel A). She was referred to our institution with concern for an ovarian tumor or ectopic pregnancy.

On admission, her Eastern Cooperative Oncology Group performance status was 0. The abdomen was distended without peritoneal signs. Speculum examination showed no abnormal bleeding or cervical findings. Transvaginal ultrasonography demonstrated a 6-cm myoma-like lesion in the anterior uterine wall and a 15×9 cm multilocular cystic pelvic mass with ascites (Figure 1, panel B). No intrauterine or extrauterine gestational sac was identified. Pelvic magnetic resonance imaging (MRI) demonstrated a 15-cm multilocular cystic ovarian tumor with focal diffusion restriction (Figure 1, panel C). Contrast-enhanced CT confirmed a large right ovarian mass with massive ascites but no evidence of lymphadenopathy or distant metastases (Figure 1, panel D). Ascitic cytology was negative for malignancy. Serum alpha-fetoprotein (AFP) was 3 ng/mL (reference range: 0-10 ng/mL), and lactate dehydrogenase (LDH) was 213 U/L (reference range: 124-222 U/L); both were within normal limits.

Because intrauterine and ectopic pregnancy were considered unlikely, an hCG-producing ovarian tumor was suspected, and elective laparotomy was performed. Preoperative serum hCG had decreased to 619.0 mIU/mL. At surgery, approximately 3,300 mL of clear yellow ascites was aspirated. The right ovary was enlarged to approximately 15 cm and impacted in the pouch of Douglas with partial adhesion to the posterior leaf of the broad ligament. The left adnexa and right fallopian tube were grossly normal. No peritoneal dissemination or enlarged lymph nodes were identified. Right salpingo-oophorectomy was performed first, and intraoperative frozen-section analysis suggested yolk sac tumor. Because fertility preservation was not desired, total abdominal hysterectomy, bilateral salpingo-oophorectomy, and partial omentectomy were completed.

**Figure 2:**
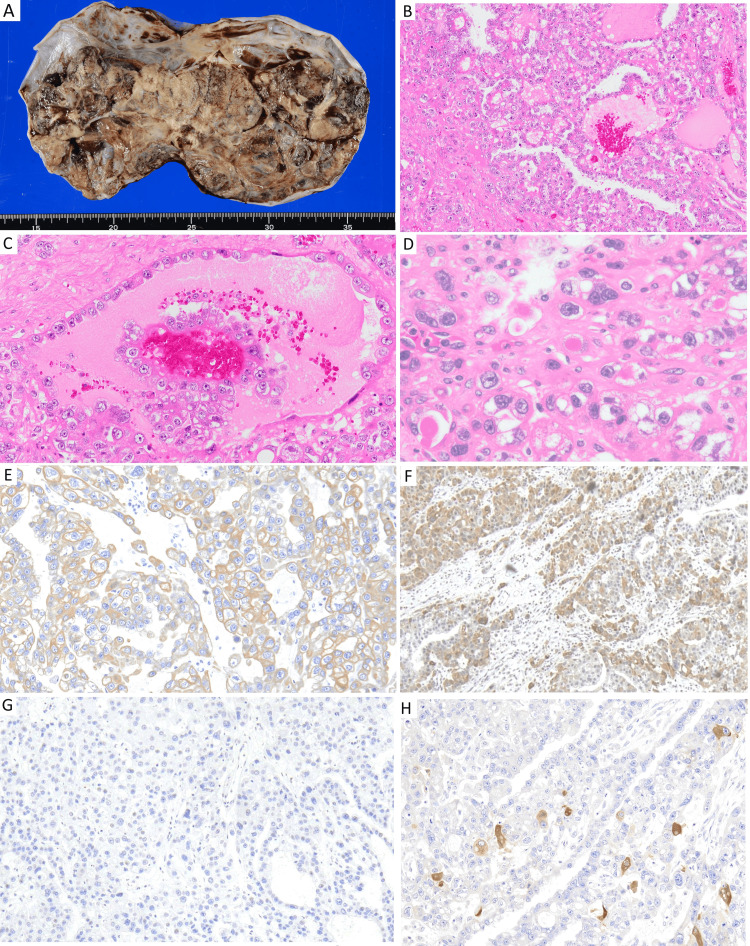
Macroscopic, histopathological, and immunohistochemical findings of the resected right ovarian tumor showing heterogeneous germ cell tumor differentiation. (A) Gross appearance of the resected tumor showing a smooth-surfaced multilocular cystic lesion with focal solid components. (B) Microscopic findings showing microcystic and papillary growth of tumor cells with clear to eosinophilic cytoplasm and enlarged round nuclei. (C) Focal Schiller-Duval body-like structure. (D) Eosinophilic hyaline globules. (E) AE1/AE3 positivity. (F) Glypican-3 positivity. (G) AFP negativity. (H) Scattered hCG-positive tumor cells.

Grossly, the tumor had a smooth external surface and a multilocular cystic cut surface with focal solid areas (Figure 2, panel A). Microscopically, tumor cells with clear to eosinophilic cytoplasm and enlarged round nuclei formed microcystic and small papillary structures (Figure 2, panel B). Focal Schiller-Duval body-like structures (Figure 2, panel C) and eosinophilic hyaline globules were present (Figure 2, panel D). Additional areas showed solid growth of highly atypical cells with pleomorphic hyperchromatic nuclei.

Immunohistochemically, tumor cells were positive for AE1/AE3 (Figure 2, panel E) and glypican-3 (Figure 2, panel F), whereas AFP, SALL4, OCT4, and CD30 were negative (Figure 2, panel G). Scattered tumor cells were positive for hCG, GATA3, and p57 (Figure 2, panel H). Placental alkaline phosphatase (PLAP) showed focal positivity, and napsin A was weakly positive in a limited area. HNF-1β was negative, and ARID1A expression was retained. The Ki-67 labeling index was approximately 30%. These findings supported an unclassifiable malignant ovarian germ cell tumor with heterogeneous differentiation but did not allow definitive subclassification under current WHO criteria [5,11].

The final diagnosis was an unclassifiable malignant ovarian germ cell tumor, International Federation of Gynecology and Obstetrics (FIGO) stage IC1 (pT1c1NXM0: primary tumor limited to one ovary with intraoperative capsule rupture {surgical spill}, regional lymph nodes not assessed, no distant metastasis), with focal choriocarcinoma-like features [12]. Adjuvant bleomycin, etoposide, and cisplatin (BEP) chemotherapy was planned. Initiation was delayed temporarily because of the exacerbation of schizophrenia. Serum hCG normalized by postoperative day 45, and four cycles of BEP were started on postoperative day 46. The patient has remained disease-free for six months since the completion of adjuvant chemotherapy.

## Discussion

This case is clinically instructive for two reasons. First, it demonstrates that some ovarian germ cell tumors remain diagnostically ambiguous despite integration of morphology, tumor markers, and immunohistochemistry. Second, it highlights a real-world management dilemma in stage I disease as follows: whether treatment intensity can be safely reduced when pathology is indeterminate and formal staging is incomplete.

The pathologic differential diagnosis included yolk sac tumor, mixed germ cell tumor with trophoblastic differentiation, and non-germ-cell mimics such as ovarian clear cell carcinoma. Focal Schiller-Duval body-like structures and hyaline globules suggested yolk sac tumor, but AFP was negative, and the overall pattern was not convincing for a dominant yolk sac component [5]. Scattered hCG-positive cells and elevated serum hCG supported trophoblastic differentiation. However, classic biphasic choriocarcinoma architecture was absent, and the trophoblastic component was only focal. Weak focal napsin A positivity raised consideration of clear cell carcinoma, but the immunophenotype with HNF-1β negativity and retained ARID1A expression argued against that interpretation [5,11].

In routine clinical care, such tumors may be best managed as malignant ovarian germ cell tumors even when exact WHO subclassification is not possible. This is particularly relevant because first-line treatment standards are derived largely from more common subtypes, whereas evidence specific to unusual, mixed, or unclassifiable ovarian primaries is limited [13-15].

A separate issue was the number of postoperative BEP cycles. Ovarian-specific studies have shown that surveillance or reduced treatment intensity can be appropriate in selected stage I patients, particularly when staging is complete, and histology is clearly favorable [6-10]. However, those de-escalation strategies are harder to justify when recurrence risk is uncertain. In the present case, the disease was confined to the ovary, and postoperative hCG normalized promptly, but formal lymph node staging was incomplete, and the tumor could not be assigned to a clearly favorable histologic category such as dysgerminoma or immature teratoma.

For that reason, treatment de-escalation was considered insufficiently supported, and four cycles of BEP were selected to prioritize oncologic safety. This decision was not driven by stage alone; rather, it reflected the combination of stage IC1 disease, incomplete formal staging, and biologic ambiguity on pathology. The durable remission observed to date supports the practical adequacy of this approach, although it does not establish superiority over a less intensive strategy. The rationale is consistent with Multicentre Italian Trials in Ovarian Cancer and Gynecologic Malignancies (MITO)-9, which identified incomplete surgical staging as a predictor of recurrence and yolk sac histology as an adverse prognostic factor [6], whereas the subsequent MITO prospective study suggested that surveillance alone is feasible only in properly staged selected patients [7].

The broader lesson is that adjuvant decision-making in ovarian germ cell tumors should account not only for stage but also for staging adequacy, confidence in histologic classification, and postoperative tumor marker behavior. When a tumor falls outside well-defined low-risk categories, the threshold for reducing treatment intensity should remain cautious. The key clinical considerations that supported four cycles of BEP in the present case are summarized in Table 1.

**Table 1:** Clinical factors supporting the choice of four cycles of BEP rather than treatment de-escalation in the present case. This table was developed by the authors of this study based on the clinical features of the present case and published evidence on stage I malignant ovarian germ cell tumor management, including the NCCN Guidelines, ESMO Clinical Practice Guidelines, and MITO studies [6-10,13]. The NCCN Guidelines recommend three cycles of BEP for good-risk disease and four cycles for poor-risk disease, but provide limited ovarian-specific detail for operationalizing this distinction in routine practice. In ovarian germ cell tumors, cycle selection is therefore commonly individualized according to disease stage, adequacy of surgical staging, histologic subtype, and postoperative tumor marker kinetics [6-10,13]. BEP: bleomycin, etoposide, and cisplatin; FIGO: International Federation of Gynecology and Obstetrics; hCG: human chorionic gonadotropin; MITO: Multicentre Italian Trials in Ovarian Cancer and Gynecologic Malignancies; NCCN: National Comprehensive Cancer Network; ESMO: European Society for Medical Oncology

Consideration	Lower-intensity approach more plausible	Reason four cycles of BEP were favored in this case
Surgical staging	Comprehensive formal staging completed	Formal lymph node staging was incomplete
Histology	Clearly favorable subtype, such as dysgerminoma or immature teratoma	Histology remained unclassifiable with focal choriocarcinoma-like features
Tumor markers	Normalization within a clearly low-risk biologic context	Serum hCG normalized, but biologic significance remained uncertain
Stage I context	Clearly low-risk stage I disease	FIGO stage IC1 plus incomplete staging and pathologic ambiguity
Overall strategy	Surveillance or reduced treatment intensity may be considered	Oncologic safety was prioritized with four cycles of BEP

## Conclusions

This case underscores the diagnostic and therapeutic challenges posed by unclassifiable malignant ovarian germ cell tumors with focal choriocarcinoma-like features. Even when definitive subclassification is not feasible, integrated interpretation of morphology, immunohistochemistry, tumor marker kinetics, and surgical findings can support a clinically useful diagnosis. In stage I disease with incomplete staging and persistent biologic uncertainty, four cycles of BEP may represent a reasonable adjuvant strategy.
